# Cerebellar Soluble Mutant Ataxin-3 Level Decreases during Disease Progression in Spinocerebellar Ataxia Type 3 Mice

**DOI:** 10.1371/journal.pone.0062043

**Published:** 2013-04-23

**Authors:** Huu Phuc Nguyen, Jeannette Hübener, Jonasz Jeremiasz Weber, Stephan Grueninger, Olaf Riess, Andreas Weiss

**Affiliations:** 1 Institute of Medical Genetics and Applied Genomics, University of Tuebingen, Tuebingen, Germany; 2 Neuroscience Discovery, Novartis Institute for BioMedical Research, Basel, Switzerland; 3 IRBM Promidis, Pomezia, Italy; UMCG, The Netherlands

## Abstract

Spinocerebellar Ataxia Type 3 (SCA3), also known as Machado-Joseph disease, is an autosomal dominantly inherited neurodegenerative disease caused by an expanded polyglutamine stretch in the ataxin-3 protein. A pathological hallmark of the disease is cerebellar and brainstem atrophy, which correlates with the formation of intranuclear aggregates in a specific subset of neurons. Several studies have demonstrated that the formation of aggregates depends on the generation of aggregation-prone and toxic intracellular ataxin-3 fragments after proteolytic cleavage of the full-length protein. Despite this observed increase in aggregated mutant ataxin-3, information on soluble mutant ataxin-3 levels in brain tissue is lacking. A quantitative method to analyze soluble levels will be a useful tool to characterize disease progression or to screen and identify therapeutic compounds modulating the level of toxic soluble ataxin-3. In the present study we describe the development and application of a quantitative and easily applicable immunoassay for quantification of soluble mutant ataxin-3 in human cell lines and brain samples of transgenic SCA3 mice. Consistent with observations in Huntington disease, transgenic SCA3 mice reveal a tendency for decrease of soluble mutant ataxin-3 during disease progression in fractions of the cerebellum, which is inversely correlated with aggregate formation and phenotypic aggravation. Our analyses demonstrate that the time-resolved Förster resonance energy transfer immunoassay is a highly sensitive and easy method to measure the level of soluble mutant ataxin-3 in biological samples. Of interest, we observed a tendency for decrease of soluble mutant ataxin-3 only in the cerebellum of transgenic SCA3 mice, one of the most affected brain regions in Spinocerebellar Ataxia Type 3 but not in whole brain tissue, indicative of a brain region selective change in mutant ataxin-3 protein homeostasis.

## Introduction

A common feature of polyglutamine diseases such as Huntington disease (HD) or the group of Spinocerebellar Ataxias (SCA), including Spinocerebellar Ataxia Type 3 (SCA3), is the formation of intranuclear aggregates in specific subtypes of neurons containing the misfolded disease protein [1]. The question if these aggregates have a toxic role in neurons is controversially discussed and so far unresolved [2], [3]. For SCA3 a relationship between processing of the disease protein ataxin-3 and disease progression was shown in tissue of transgenic mice and SCA3 patients where an increasing amount of ataxin-3 fragmentation was linked to disease severity [4]. Very recently, different studies revealed a proteolytic cleavage of mutant ataxin-3 by calpains [5]–[7] which results in the formation of highly aggregation-prone polyQ-containing fragments [5]. Therefore, analysis of soluble mutant ataxin-3 thus offers potential for evaluating the efficacy of possible therapeutic agents or as a biomarker for SCA3 disease progression.

Biomarker research in the field of neurodegenerative diseases gained increased interest in the last years due to the difficult monitoring and heterogeneous clinical nature of these disorders in which disease progression likely occurs over decades prior to appearance of first clinical symptoms. Blood- or cerebrospinal fluid-based biomarkers in neurodegenerative diseases are thus of major importance in context of disease risk prediction, improvement of diagnosis and prognosis, and optimization of therapeutic studies. In part, application of biomarker diagnostic in the clinical routine depends on the establishment of novel technologies in this field [8].

As aggregation of disease causing proteins is a hallmark in post mortem brains of patients with neurodegenerative diseases, quantification of their soluble and aggregated conformations are potential useful readouts for biomarker development [9]. Time-resolved Förster resonance energy transfer (TR-FRET) immunoassays have been shown to be a robust and reliable method for detection of soluble and aggregated mutant huntingtin, the disease protein in HD, in a high spectrum of biological material including cellular, animal and human samples [10]. In addition, the quantification of the soluble and aggregated disease protein species using similar TR-FRET immunoassay based analyses is currently being investigated as potential biomarkers in Alzheimer disease (AD) [11] and Parkinson disease (PD) [12], [13]. In the last months different groups implemented and modified this technique to also analyze the ratio between soluble and aggregated disease protein [9] or the ratio of oligomerization of proteins [13], [14].

We thus chose to investigate levels of soluble mutant ataxin-3 during disease progression in brain tissue of SCA3 transgenic mice. In summary, we were able to develop a new robust, reproducible and sensitive TR-FRET immunoassay to analyze the level of soluble polyglutamine-expanded ataxin-3 in cellular and animal tissue and found that soluble mutant ataxin-3 levels show a tendency for decrease specifically in the cerebellum, one of the most affected brain regions in SCA3.

## Materials and Methods

### Ethics Statement

This study was carried out in strict accordance with the recommendations in the Guide for the Care and Use of Laboratory Animals of the University of Tuebingen, Germany. The protocols were approved by Institutional Animal Care and Use Committee (IACUC) of the University of Tuebingen.

### Antibodies

In this study two different antibodies (1H9 and MW1) were used, both bind to the ataxin-3 protein. 1H9 antibody (MAB5360, Millipore) recognizes a 20 amino acid polypeptide 63 amino acids upstream of the disease causing polyglutamine tract (epitope E214 to L233) and the MW1 antibody, which binds specifically elongated and mutated polyQ-stretches. The MW1 antibody was developed by Paul Patterson [15] and obtained from the Developmental Studies Hybridoma Bank developed under the auspices of the NICHD and maintained by The University of Iowa, Department of Biological Sciences, Iowa City, IA 52242.

### Cellular Model

HEK293T cells were grown in Dulbeccós modified eagle medium supplemented with 10% fetal calf serum, 1% non-essential amino acids and 1% penicillin/streptomycin at 37°C in 5% CO_2_. Cells were plated in 96-well tissue culture plates, immediately transfected with V5-tagged ataxin-3 constructs (containing 15, 77 and 148 CAG repeats) using Attractene transfection reagent according to the manufacturer’s instructions (Qiagen) and subsequently incubated for 24 h.

### Animal Model

The generation and first characterization of the SCA3 transgenic mouse model used here has been described previously [16]. Briefly, a full-length ataxin-3 construct (isoform c) containing 70 CAG repeats under the control of a 3.4 kb fragment of the murine prion protein (Prp) promoter was used to generated SCA3 transgenic mice. The mice developed a severe neurological phenotype with gait ataxia and tremor beginning at the age of 10 months [16].

### Sample Preparation

Brain samples were generated by sonication of adult mouse brains (whole brains or cerebellum) in 10x v/w of ice-cold lysis buffer (PBS +1% TritonX100+1X protease inhibitor cocktail (Roche)). HEK293T cell lysates were prepared by removal of cell culture medium and addition of lysis buffer to the adherent cells. Cells were lysed for 30 min under 300 rpm at 4°C. Total protein concentration was quantified by BCA assay (Pierce).

### Time-resolved FRET Immunoassay

1H9 was labeled with donor Lumi4-Tb-fluorophore (Cisbio). MW1 was labeled with D2 acceptor fluorophore (Cisbio). After optimization of antibody titers and incubation conditions, quantification of mutant ataxin-3 levels was performed in low volume polystyrene 384 microtiter plates (Greiner Bio-One) with 5 µl sample volume and addition of 1 µl antibody solution (50 mM NaHPO_4_+400 mM NaF +0.1% BSA +0.05% Tween-20+0.3 ng/µl 1H9-Tb +10 ng/µl MW1-D2). Plates were then incubated at 4°C for 20 h and analyzed by time-resolved fluorescence at 620 nm and 665 nm on an Envision Multilabel reader (Perkin Elmer).

### Immunohistochemistry

For aggregate analysis, brains of 12 and 22 months old mice (3 of each genotype) were fixed by perfusion with 4% paraformaldehyde. Afterwards, 7 µm sections of paraffin-embedded brain tissue were processed and stained as previously described [16]. Primary antibody (ataxin-3, clone 1H9, Millipore) binding was performed in a 1∶4000 dilution overnight at 4°C. Antibody binding was visualized by peroxidase labeling using vectastain elite avidin and biotinylated enzyme complex kit with 3,3′-diaminobenzidine substrate following the manufactureŕs instructions. Quantification of number and size of ataxin-3 positive inclusions was performed by analyzing ten visual fields (spread from lobule 6 to 10) on three different sections per analyzed mouse using an x63 objective on a Zeiss Axiovert image microscope. Image acquisition and quantification of number and size of aggregates in the granular layer of the cerebellum was done blinded using ImageJ analysis software (National Institutes of Health, NIH). The average number and size of inclusions was calculated for each animal and data are presented as the mean ± standard deviation.

### Immunofluorescence Double Staining

Calbindin immunostaining of 3 mice per genotype and age was used to analyze the progression of neuronal dysfunction in the cerebellum of SCA3 transgenic mice compared to wildtype littermates. For double-fluorescence staining, 7 µm sections of paraffin-embedded brain tissue were incubated with the polyclonal anti-calbindin D-28K antibody (1∶1000 Swant Swiss antibodies) and monoclonal anti-ataxin-3 antibody (clone 1H9; 1∶500; Millipore) overnight at 4°C, followed by incubation with the corresponding fluorescence-coupled secondary antibodies (rabbit anti-Cy2 (1∶50) and mouse anti-Cy3 (1∶100); both from Dianova). Cell nuclei were stained with the fluorescent chromatin dye 4',6-diamidino-2-phenylindole (DAPI).

Quantification of calbindin immunoreactivity was performed by screening 10 fields in the cerebellum (lobule 6 to 10) on three different sections per analyzed mouse using an x40 objective on a Zeiss Axiovert image microscope. Optical densitometry analysis was done using ImageJ analysis software. Values are presented as the mean value of calbindin optical density per analyzed field ± standard deviation (u.a. = arbitrary unit).

### Western Blot

Western Blot analyses were carried out as previously described [16]. Immunodetection was performed using following antibodies: monoclonal mouse anti-ataxin-3 antibody (1∶2500, clone 1H9, Millipore), monoclonal mouse anti-β-actin antibody (1∶10,000, clone AC-15, Sigma), HRP-conjugated goat anti-mouse antibody (1∶2500, GE Healthcare).

### AGERA

AGERA (agarose gel electrophoresis for resolving aggregates) method and isolation of cytoplasmic and nuclear fractions were performed as previously described [17]. Cytoplasmic and nuclear fractions of brain samples from 6 months old mice (wildtype, SCA3 transgenic, R6/2) were run on a long 1% agarose gel and immunoblotted with specific antibodies for both ataxin-3 (clone 1H9, MAB5360, Millipore) and huntingtin (MW8).

### Data Analysis

Unless otherwise indicated, TR-FRET signals are presented as ΔF values, which normalize the emission of the TR-FRET-dependent signal of the D2 acceptor fluorophore (665 nm) to that of the FRET-independent Terbum-cryptate donor fluorophore (620 nm) after subtracting the assay buffer background fluorescence. This method takes advantage of the internal reference fluorescence intensity of the donor fluorophore, thereby providing a HTT protein signal corrected for potential assay-interfering artifacts such as turbidity, light scattering or quenching capability of the sample, differences in sample volume due to slight pipetting variability, and day-to-day assay fluctuation caused by differences in excitation lamp energy. As a final step, the percentage TR-FRET signal increase over background is then normalized to total protein content of the sample as determined by BCA assay.

### Statistical Analysis

All data were analyzed using Prism 6.0 software, GraphPad. Standard two-way ANOVA (data not matched) to assess the effects of genotype and age and Bonferroni *post hoc* tests were conducted to compare individual genotype effects at individual ages. Data are presented as mean ± SEM. Differences were considered significant if *p*<0.05.

## Results and Discussion

### Establishing a TR-FRET based Immunoassay to Detect Soluble Ataxin-3 Levels

**Figure 1:**
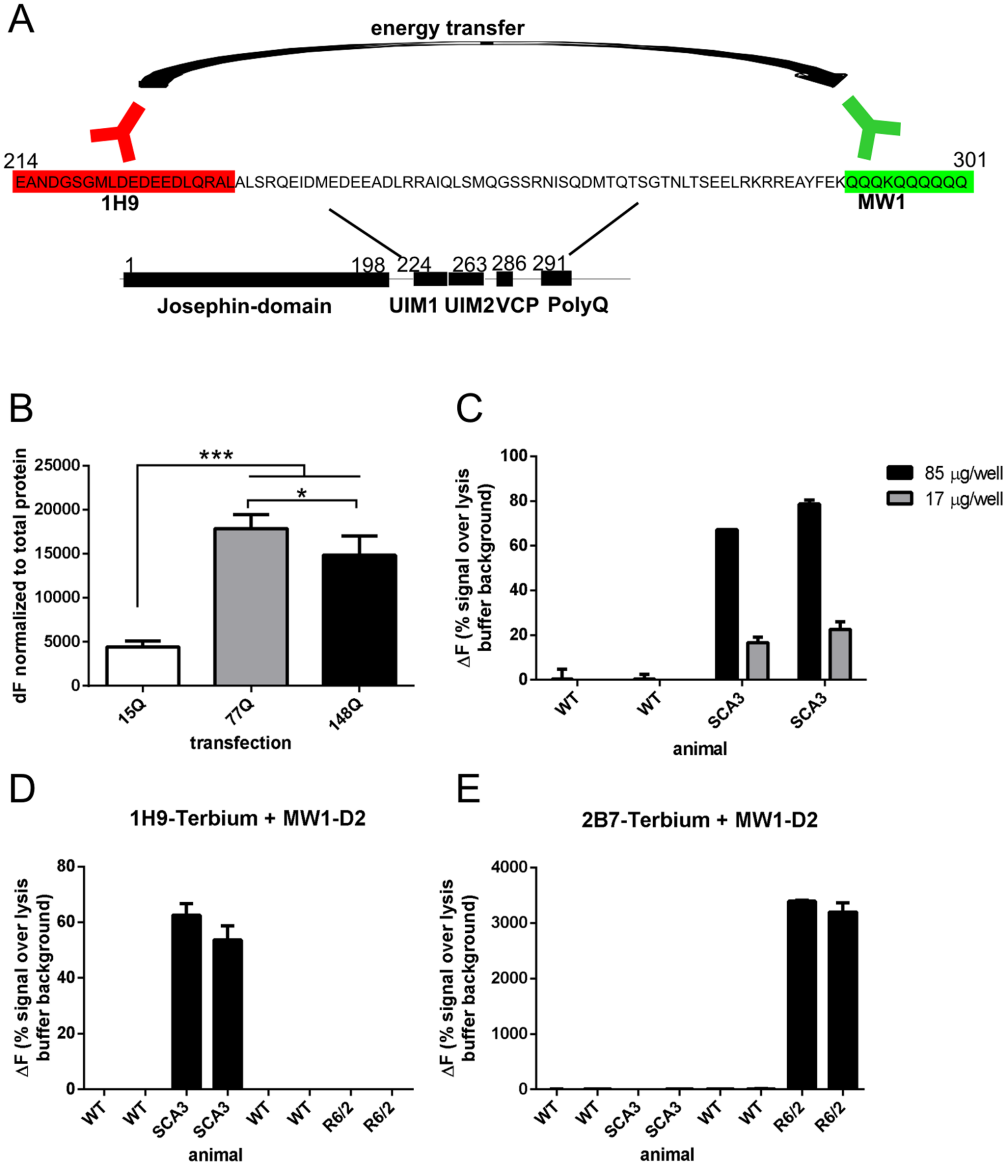
Establishing a TR-FRET based immunoassay to detect soluble ataxin-3 levels. A) Schematic illustration of antibody binding sites in context of the ataxin-3 protein and the principal of the TR-FRET immunoassay. By using this antibody combination (1H9 and MW1) only ataxin-3 with more than 6 CAG repeats can be detected (no detection of wildtype mouse ataxin-3) since the MW1 antibody is specific against an expanded polyQ-stretch. B) Measuring mutant ataxin-3 levels in HEK293T cells transiently transfected with ataxin-3 with different polyQ-lengths (15Q, 77Q, 148Q) revealed a detection of mutant ataxin-3 with 15, 77 and 148 polyglutamine repeats. Furthermore, ataxin-3 with a highly elongated polyQ-repeat (77 or 148Q) can be detected more efficiently than ataxin-3 with a normal, non-expanded repeat (15Q, ***p<0.001). Formation of aggregates was only found in cells transfected with ataxin-3-148Q but not with ataxin-3-77Q, which is associated with a significant reduction of soluble ataxin-3-148Q compared to ataxin-3-77Q (*p<0.05). C) To validate detectable protein amounts in mouse brain homogenates different concentrations of protein levels were tested. Whereas in wildtype lysates only a background noise signal was measured, in SCA3 transgenic whole brain lysates a protein concentration dependent TR-FRET signal was detected. D, E) To validate the specificity of the used antibody combinations for the disease protein ataxin-3 (1H9– MW1) or huntingtin (2B7– MW1) a detection of protein in ataxin-3 transgenic mice (SCA3), huntingtin transgenic mice (R6/2) and wildtype mice was carried out. The antibody combination for ataxin-3 (1H9– MW1) showed only a specific signal in SCA3 transgenic mice, whereas the antibodies tailored for huntingtin (2B7– MW1) only detect in R6/2 huntingtin transgenic mice. Bars represent averages and standard error of the mean n = 6.

To investigate the levels of soluble mutant ataxin-3 during disease progression, we aimed to develop a robust and easy-to-use TR-FRET immunoassay suitable for analysis of cellular and animal samples. To achieve this aim, we focused on the immunoassay antibody pair 1H9 and MW1, which should specifically bind to mutant but not wildtype ataxin-3. Higher specificity for the mutant form is a direct result of both antibodies binding in proximity to each other with the MW1 antibody recognizing the elongated and mutated polyglutamine region [15], whereas the 1H9 antibody recognizes a sequence 58 amino acids N-terminal to the MW1 (schematic illustration in Fig. 1A). Prior to in-depth analysis of biological material we optimized the assay protocol for lysis buffer conditions (Sup. Fig. S1), the donor:acceptor antibody titers and incubation conditions (Sup. Fig. S2). Following optimization, all analyzed samples were subsequently lysed in PBS buffer supplemented with 1% TritonX100 and incubated with 0.3 ng/µl 1H9-Tb and 10 ng/µl MW1-D2 at 4°C for 20 h.

Next, we transfected HEK293T cells with ataxin-3 constructs with different polyglutamine length (15Q, 77Q and 148Q) and monitored the expression level of the respective ataxin-3 using the TR-FRET immunoassay. As expected, ataxin-3 with a highly elongated polyglutamine repeats (77 or 148Q) can be detected more efficiently than ataxin-3 with a normal non-expanded repeat (15Q; p<0.001, Fig. 1B). The most frequent non-expanded allele found in normal individuals is 14 glutamines [18]. While our analysis revealed that the wildtype length can also be detected by the assay, the sensitivity for mutant over wildtype proved to be highly increased. Additionally, overexpressing human ataxin-3 with 148 or 77 glutamines in HEK293T cells results in aggregate formation only in cells transfected with ataxin-3-148Q but not in cells transfected with ataxin-3-77Q (data not shown). Notably, this is associated with a significant reduction of soluble ataxin-3-148Q compared to levels of ataxin-3-77Q as detected by TR-FRET analyses (Fig. 1B, p<0.05).

We then proceeded to assess mutant ataxin-3 detection in crude brain homogenates of 6 months old wildtype and SCA3 transgenic mice overexpressing human mutant ataxin-3. Mutant ataxin-3 could be readily quantified with high specificity over endogenous wildtype ataxin-3 in these samples (Fig. 1C). Further, to validate the specificity of the used antibody combinations for mutant ataxin-3 over other mutant polyglutamine containing proteins such as mutant huntingtin we tested the specificity in brain lysates of SCA3 and R6/2 mice, the most commonly used HD mouse model [19]. As expected, the antibody combination 1H9 and MW1 shows a mutant ataxin-3 specific signal in SCA3 transgenic mice, whereas the antibodies 2B7 and MW1 specifically bind to mutant huntingtin in transgenic Huntington mice (Fig. 1D, E).

### Ataxin-3 Detection in Cerebellar Samples Reveals a Tendency for Decrease in Soluble polyQ-expanded Ataxin-3 during Disease Progression

After establishing the assay for ataxin-3 we analyzed if the assay is suitable to measure disease progression in tissue samples from transgenic SCA3 mice. Therefore, we monitored the level of soluble mutant ataxin-3 with 70 glutamines in whole brain homogenates or cerebellar lysates from transgenic SCA3 mice [16] at the age of 12 and 22 months compared to age- and sex-matched wildtype controls.

**Figure 2:**
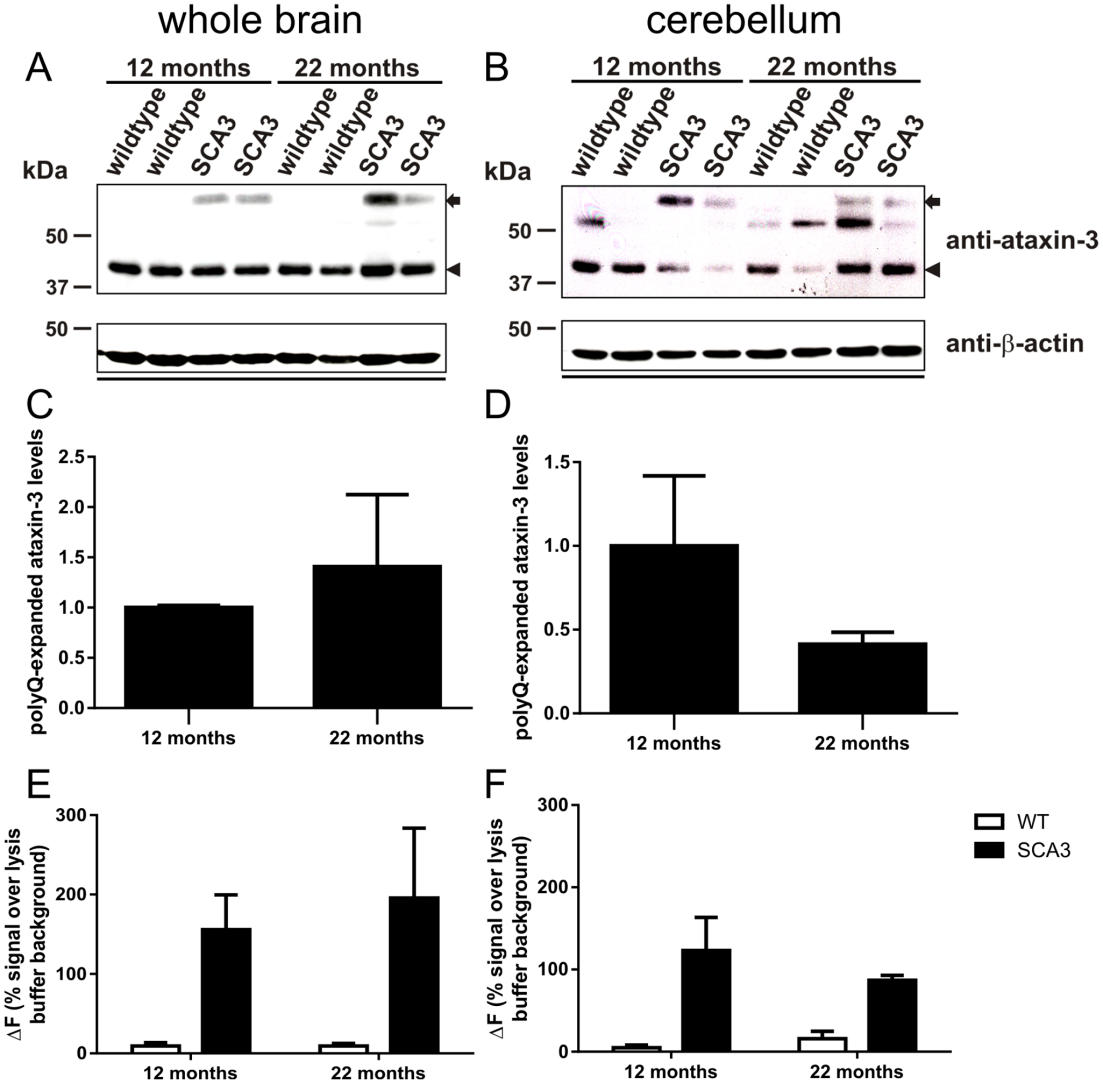
Western blot and TR-FRET analyses revealed an age dependent decrease of soluble mutant ataxin-3 levels in cerebellum. A–D) Two animals of the indicated genotypes per age were immunoblotted and detected with an ataxin-3 (clone 1H9) antibody. In all samples the endogenous ataxin-3 at 42 kDa was detected (indicated by an arrow head). In transgenic SCA3 mice a protein band at 60 kDa revealed the human ataxin-3 protein with 70Qs (arrow). Whole brain lysates showed similar expression levels of overexpressed human ataxin-3 in SCA3 transgenic mice at the age of 12 and 22 months (A and densitometric analysis in C; p = 0.6). In the cerebellum, one of the mainly affected brain areas in SCA3, less overexpressed ataxin-3 is detectable at the age of 22 months compared to 12 months of age (B). Densitometric analysis confirmed this observation (D, p = 0.3). As loading control actin is shown. E, F) Analysis of these samples by TR-FRET detection revealed similar levels of ataxin-3 in SCA3 transgenic mice in whole brain lysates at the age of 12 and 22 months (p = 0.52; E). In comparison in homogenates of the cerebellum the level of overexpressed ataxin-3 in SCA3 transgenic mice decreases in an age dependent manner, although this did not reach statistical significance (p = 0.19, F). Bars represent averages and standard deviation of biological triplicates.

**Figure 3:**
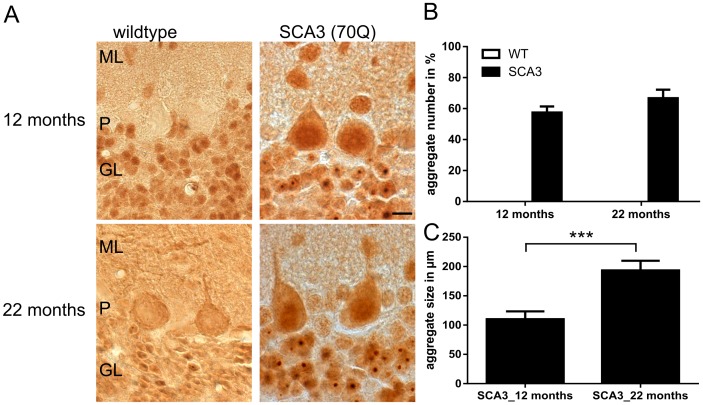
The number of ataxin-3 positive aggregates is inversely correlated with the level of soluble ataxin-3 in Western blot and TR-FRET analyses. A) shows representative immunohistochemical staining with an antibody against ataxin-3 (clone 1H9) in SCA3 transgenic mice compared to sex- and age-matched wildtype controls at the age of 12 and 22 months. No aggregates are detectable in wildtype animals. However, in SCA3 transgenic mice increasing numbers of aggregates are found at the age of 22 months compared to 12 months (A), but this did not reach significance (p = 0.076) after counting three independent animals per genotype (B). C) Measuring the size of aggregates in the granular layer of the cerebellum on the other hand revealed significant larger aggregates with disease progression in SCA3 transgenic mice (***p<0.001). Scale bar = 20 µm, ML = molecular layer, P = Purkinje cells and GL = granular layer.

**Figure 4:**
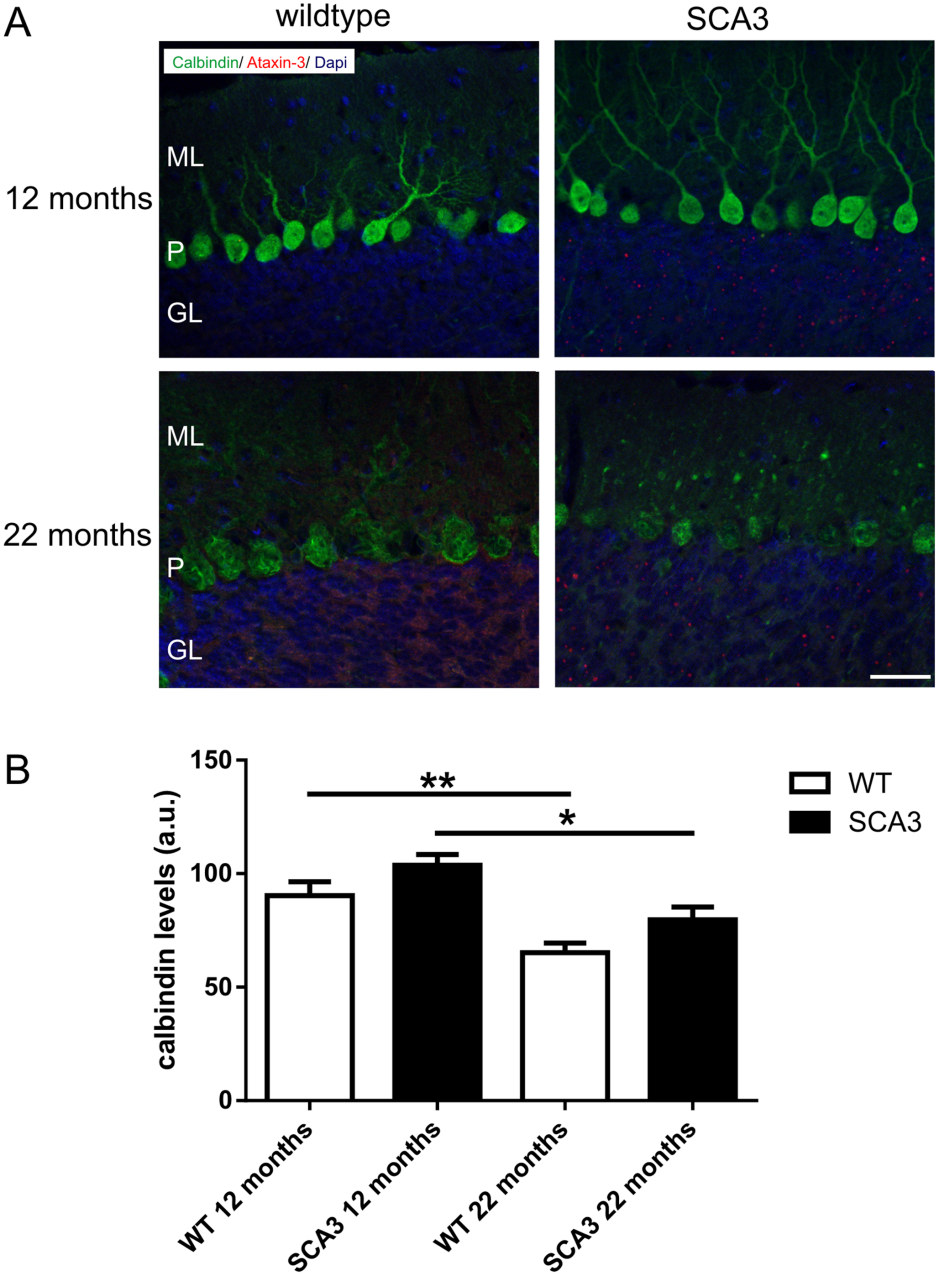
Calbindin immunoreactivity showed reduced arborization of Purkinje cells with age independently from transgene expression. A) Double-immunofluorescence staining with calbindin (green) and ataxin-3 (clone 1H9, red) revealed aggregates in the granular layer of the cerebellum but not in the Purkinje cell layer of SCA3 transgenic mice. Calbindin staining of the Purkinje cells demonstrated shrinkage and loss of cells as well as a reduction of arborization of Purkinje cells with age in both, wildtype and SCA3 transgenic mice. B) Quantification of optical densitometry of calbindin showed a loss of immunoreactivity with age in both genotypes, respectively (*p<0.05; **p<0.01; a.u. = arbitrary units). Scale bar = 20 µm, ML = molecular layer, P = Purkinje cells and GL = granular layer.

As represented by Menzies et al., (2010) the transgenic SCA3 mice with 70 glutamines (line 70.61, Ref. 16) did not longer demonstrate as severe a phenotype as previously reported [20]. Now, SCA3 transgenic mice developed first neurological symptoms including gait ataxia and tremor at the age of ten months, which progressively worsen with weight loss and result in a premature death at the age of 22 to 23 months. Using rotarod performance to measure motor coordination in these mice demonstrated a decreased ability to walk starting at the age of 10 weeks [20]. Immunohistochemical staining demonstrated first aggregates at the age of 3 months. In a first step, to measure a possible reduction of soluble mutant ataxin-3 with disease progression we analyzed whole brain lysates on Western blot (Fig. 2A, C) and by TR-FRET (Fig. 2E) and found similar levels of soluble ataxin-3 at the age of 12 and 22 months (p = 0.62 for Western blot analyses and p = 0.52 for TR-FRET). In contrast, in one of the main pathogenesis areas of SCA3, the cerebellum [21], both techniques revealed less soluble ataxin-3 at the age of 22 months compared to 12 months of age (Fig. 2B, D, F), although this decrease did not reach statistical significance (Western blot: p = 0.3; TR-FRET: p = 0.19). In both TR-FRET analyses, whole brain and cerebellum, the specific signal of polyQ-expanded ataxin-3 was up to 200% higher than in wildtype mice, which demonstrated that the endogenous mouse ataxin-3 with around 6 glutamines is nearly undetected with this method (Fig. 2E, F). A reduction of soluble levels of mutant ataxin-3 in the cerebellum could possibly be an effect of increased aggregate formation with disease progression in these transgenic mice. Therefore, we analyzed the aggregate load in cerebellar slices of transgenic SCA3 mice at the age of 12 and 22 months by immunohistochemical staining using 1H9 antibody. As described earlier, in wildtype controls no aggregates were detectable [16]. However, in SCA3 transgenic mice up to 70% of granular cells in the cerebellum showed aggregates in the nucleus, whereas at the age of 12 months less aggregates (up to 55%) were found (Fig. 3A). A quantification of the aggregate load demonstrated a tendency of an increased number of aggregates in cerebellar neurons at the age of 22 months, but this did not reach significance after counting three independent transgenic animals per genotype (Fig. 3B, p = 0.07). Additionally, the quantification of the aggregate size revealed significant larger aggregates at the age of 22 months compared to 12 months of age (Figure 3C, p<0.001). Presence of nuclear, but also cytoplasmic aggregates in SCA3 transgenic mouse brains was further validated with AGERA (Sup. Fig. S3). To investigate the association of aggregate formation and neuronal degeneration in the cerebellum of SCA3 transgenic mice we performed double immunofluorescence staining with calbindin (green signal) and ataxin-3 antibodies (clone 1H9, red signal). Similar to the immunohistochemistry data (Figure 3) staining with ataxin-3 revealed a higher aggregate load in 22 months old SCA3 transgenic mice compared to 12 months old SCA3 mice. Furthermore, staining with calbindin demonstrated shrinkage and loss of Purkinje cells as well as a reduced arborization of these cells with age in both wildtype and SCA3 transgenic mice (Figure 4A, B).

In summary, with the combination of the techniques applied (Western blot, immunohistochemistry and TR-FRET) we confirmed the hypothesis that the level of soluble polyQ-expanded ataxin-3 in the cerebellum of transgenic mice decreases with disease progression, whereas the aggregate load in the same brain region inversely correlates with this observation. Similar results were found in Huntington disease mice (R6/2) where also an inverse correlation between the level of soluble mutant huntingtin and aggregate formation was observed [9], [22]. These findings demonstrate that in these two distinct polyglutamine diseases, Huntington disease and SCA3, the soluble level of the disease protein correlates with disease progression and, therefore, possibly representing a biomarker for these disorders. In both, HD and SCA3, it is known that the disease protein has to be proteolytically cleaved by caspases and/or calpains before the formation of nuclear aggregates occurs. The cleavage separates the expanded polyglutamine tract from the rest of the protein, subsequently allowing the polyQ-tract to translocate to the nucleus and form intranuclear aggregates. For huntingtin, the cleavage sites of caspases [23]–[26] and calpains [27], [28] are well known. However for ataxin-3, there is an ongoing discussion whether caspases [4], [23], [29]–[31] or calpains [5]–[7] can cleave ataxin-3. Very recently, it was convincingly shown that calpains can proteolyze ataxin-3 [5]–[7] and different cleavage sites have been proposed [32]. Therefore, in another study we utilized our novel TR-FRET immunoassay to investigate whether a hypothetical cleavage site around amino acid position 260 [32] in the ataxin-3 protein, which is located between the both antibodies used for TR-FRET, is indeed a cleavage site for calpains. To this end, we treated cerebellar lysates of SCA3 transgenic mice with appropriate calcium levels to activate calpain-1 or calpain-2. Under these conditions we found that the TR-FRET signal is decreased by half compared to the native, untreated ataxin-3 signal [6] indicating that decreased detection of soluble mutant ataxin-3 after calcium induction was due to increased calpain cleavage at amino acid position 260. This demonstrates that our TR-FRET immunoassay cannot only be applied to measure the level of soluble mutant ataxin-3 but by use of specific antibody combinations it can be of great value to investigate specific pathomechanisms such as identifying cleavage sites of ataxin-3.

### Conclusions

In summary, we described the adaptation of a new robust, specific, reproducible and sensitive time-resolved Förster resonance energy transfer (TR-FRET) immunoassay to analyze the level of soluble polyglutamine-expanded ataxin-3 in cellular and animal tissue. Using this assay we were able to detect a tendency for a decrease of soluble polyQ-expanded ataxin-3 in mouse brain lysates, particularly in the cerebellum, with disease progression. In line with this observation, we found more nuclear aggregates in the respective brain region and therefore described an inverse correlation between level of soluble ataxin-3 and aggregate formation.

To identify biomarkers of neurodegenerative diseases a simple and sensitive assay is needed. As shown for AD, HD, and PD, the TR-FRET immunoassay is a technique, which can be very useful for this implement. Therefore, the adaptation of the assay to SCA3 will help to understand pathomechanisms, monitor disease progression in different patient material (e.g. blood, cerebrospinal fluid) and help to evaluate the efficiency of therapeutic strategies.

## Supporting Information

Figure S1 **Determination of optimal lysis buffer composition for mutant ataxin-3 extraction and subsequent TR-FRET detection.** Wildtype and SCA3 transgenic mouse brain were homogenized with the indicated lysis buffers. Homogenates were adjusted to identical total protein concentrations and subjected to TR-FRET detection.
(TIF)

Figure S2 **Optimization of donor:acceptor antibody ratios and kinetics for TR-FRET detection of mutant ataxin-3.** SCA3 transgenic mouse brain homogenate was analyzed with different antibody titers and assay incubation conditions. Samples were incubated at either room temperature or at 4°C for the indicated duration. Incubation for 22 h at 4°C with 0.3 ng/well 1H9-Terbium and 3 ng/well MW1-D2 antibody yielded the maximum signal over background window. These conditions were subsequently used for further analysis of biological samples in this report. Bars represent averages and standard deviation of n = 3.
(TIF)

Figure S3 **Analysis of ataxin-3 aggregates in mouse brain by AGERA.** Representative example of an AGERA blot of mouse brain samples from ataxin-3 mice with 70 glutamines compared to wildtype controls and Huntington transgenic R6/2 mice. Both ataxin-3 transgenic as well as R6/2 transgenic mice showed aggregates in the cytoplasmic (C) and nuclear (N) fraction using specific antibodies for both ataxin-3 (1H9) and huntingtin (MW8). In the R6/2 mice aggregates are significantly larger than in SCA3 transgenic mice. No aggregates were found in wildtype mice.
(TIF)
