# Supplementary figures and images for: Cerebellar Soluble Mutant Ataxin-3 Level Decreases during Disease Progression in Spinocerebellar Ataxia Type 3 Mice
Source: PLoS One. 2013 Apr 23;8(4):e62043. doi: 10.1371/journal.pone.0062043 (PMC3633920; doi:10.1371/journal.pone.0062043)

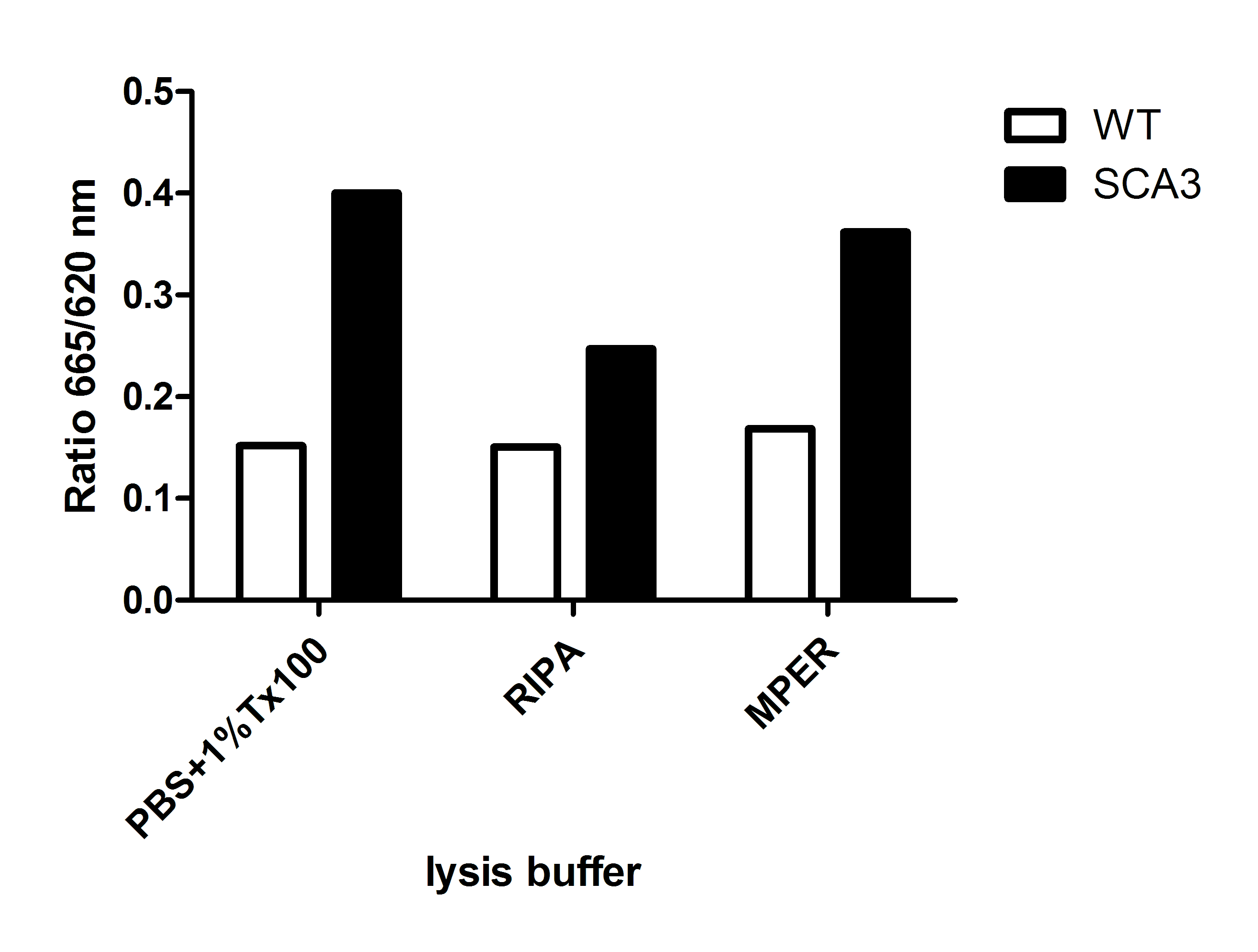

Supplement: Figure S1 — Determination of optimal lysis buffer composition for mutant ataxin-3 extraction and subsequent TR-FRET detection. Wildtype and SCA3 transgenic mouse brain were homogenized with the indicated lysis buffers. Homogenates were adjusted to identical total protein concentrations and subjected to TR-FRET detection. (TIF) [file pone.0062043.s001.tif]

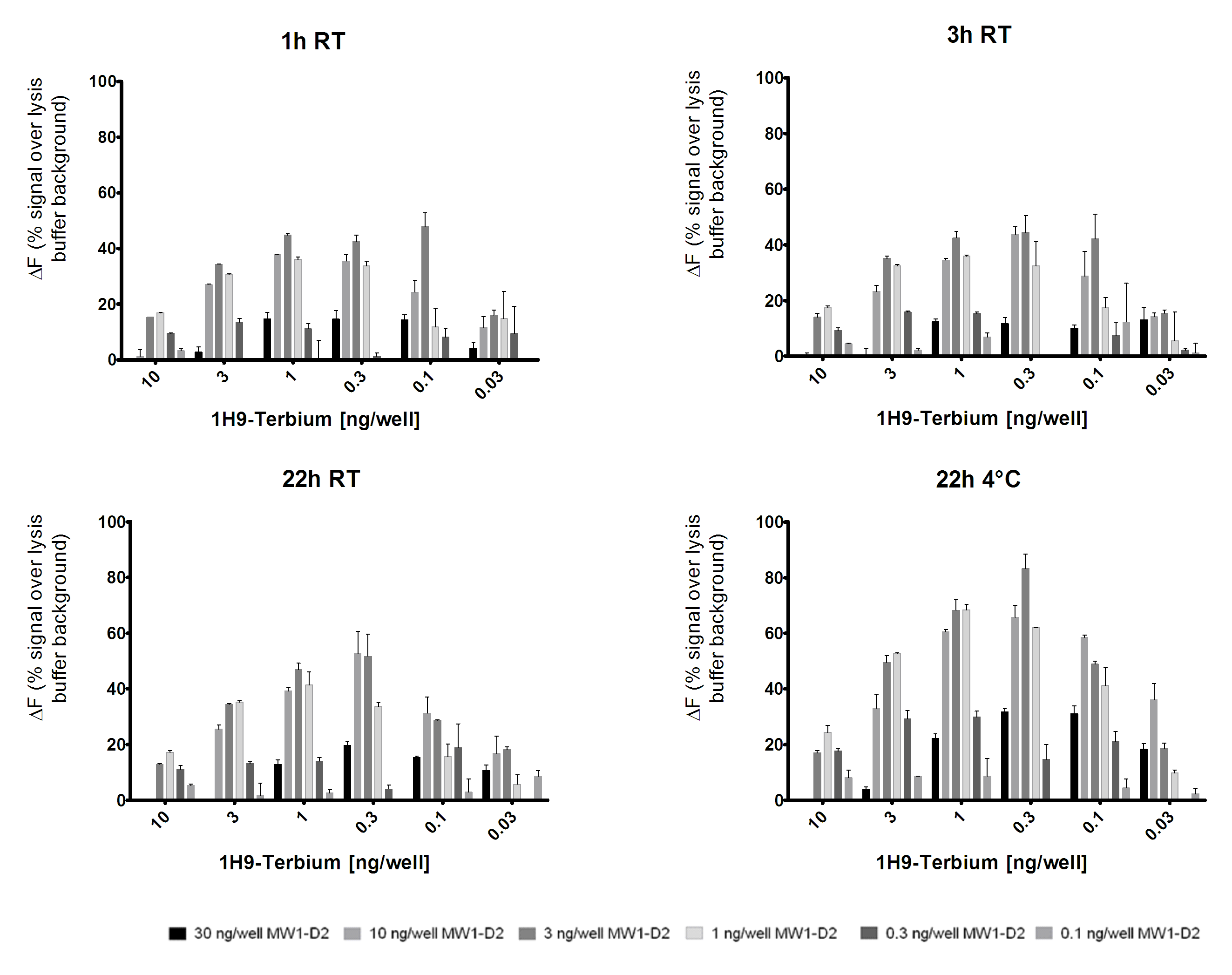

Supplement: Figure S2 — Optimization of donor:acceptor antibody ratios and kinetics for TR-FRET detection of mutant ataxin-3. SCA3 transgenic mouse brain homogenate was analyzed with different antibody titers and assay incubation conditions. Samples were incubated at either room temperature or at 4°C for the indicated duration. Incubation for 22 h at 4°C with 0.3 ng/well 1H9-Terbium and 3 ng/well MW1-D2 antibody yielded the maximum signal over background window. These conditions were subsequently used for further analysis of biological samples in this report. Bars represent averages and standard deviation of n = 3. (TIF) [file pone.0062043.s002.tif]

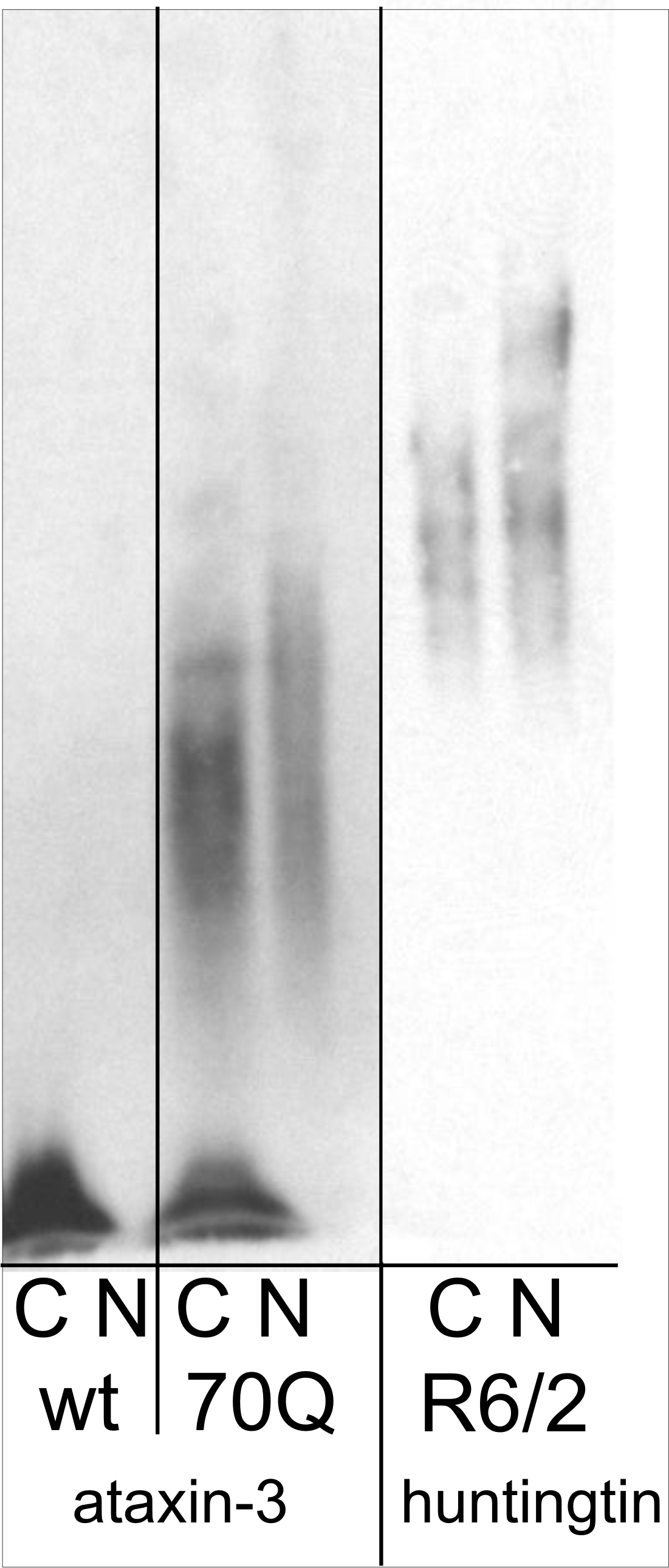

Supplement: Figure S3 — Analysis of ataxin-3 aggregates in mouse brain by AGERA. Representative example of an AGERA blot of mouse brain samples from ataxin-3 mice with 70 glutamines compared to wildtype controls and Huntington transgenic R6/2 mice. Both ataxin-3 transgenic as well as R6/2 transgenic mice showed aggregates in the cytoplasmic (C) and nuclear (N) fraction using specific antibodies for both ataxin-3 (1H9) and huntingtin (MW8). In the R6/2 mice aggregates are significantly larger than in SCA3 transgenic mice. No aggregates were found in wildtype mice. (TIF) [file pone.0062043.s003.tif]
